# Draft Genome Sequence of the Plant Growth–Promoting Rhizobacterium *Acinetobacter radioresistens* Strain SA188 Isolated from the Desert Plant *Indigofera argentea*

**DOI:** 10.1128/genomeA.01708-16

**Published:** 2017-03-02

**Authors:** Feras F. Lafi, Intikhab Alam, Ton Bisseling, Rene Geurts, Vladimir B. Bajic, Heribert Hirt, Maged M. Saad

**Affiliations:** a Biological and Environmental Sciences and Engineering Division (BESE), King Abdullah University of Science and Technology (KAUST), Thuwal, Kingdom of Saudi Arabia; b Computational Bioscience Research Center (CBRC), King Abdullah University of Science and Technology (KAUST), Thuwal, Kingdom of Saudi Arabia; c Department of Plant Sciences, Laboratory of Molecular Biology, Wageningen University, Wageningen, The Netherlands

## Abstract

*Acinetobacter radioresistens* strain SA188 is a plant endophytic bacterium, isolated from root nodules of the desert plants *Indigofera* spp., collected in Jizan, Saudi Arabia. Here, we report the 3.2-Mb draft genome sequence of strain SA188, highlighting characteristic pathways for plant growth–promoting activity and environmental adaptation.

## GENOME ANNOUNCEMENT

In an effort to explore the microbial diversity of desert pioneer plants, the Darwin21 project (http://www.darwin21.net) has been established. In the framework of the project, an extensive collection of microbial isolates from roots of different desert plants has been conducted. Preliminary results revealed a large diversity of bacterial species with a potential to promote the growth of *Arabidopsis thaliana* plants under different biotic and abiotic stresses. A selected number of these strains was sequenced and characterized as described previously (1, 2). *Acinetobacter radioresistens* strain SA188 is an endophytic bacterium isolated from surface-sterilized root nodules on roots of the pioneer plant *Indigofera argentea* Burm.f. (*Fabaceae*). Plants were collected from different regions in the Jizan area (16°56.475′N, 42°36.694′E) of the Kingdom of Saudi Arabia. Based on the 16S rRNA gene sequence, strain SA188 is closely related (with 99% genome similarity) to *A. radioresistens* strain NBRC 102413 (NR_114074.1) and to *A. radioresistens* strain FO-1 (NR_026210) (1).

The genomic DNA of strain SA188 was extracted using Qiagen’s DNeasy blood and tissue kit following the manufacturer’s protocol. The DNA was then sequenced using paired-end Illumina MiSeq, and the library preparation was constructed as described previously (2). Contig assembly was done with Spades assembler version 3.6 (3) with a 1-kb contig cutoff size. *De novo* assembly of MiSeq reads for *A. radioresistens* strain SA188 resulted in 38 contigs with a total length of 3,208,318 bp and a mean contig size of 84,429 bp. The *N*_50_ was 289,666 bp, the L50 was reached with four contigs, and the G+C content was 41.5%. MegaBLAST (4) searches of strain SA188 concatenated contigs against the NCBI reference genome database (http://www.ncbi.nlm.nih.gov/genome) revealed that the closest relative was *A. radioresistens* strain NBRC 102413, with top hits for three scaffolds (NZ_KB849748.1, NZ_KB849749.1, and NZ_KB849747.1) at coverages of 16%, 56%, and 14% (86% accumulative coverage) and sequence identities of 98% to 99%. The annotation of strain SA188 was carried out using the default INDIGO pipeline (5) with the exception of the open reading frame (ORF) prediction by FragGeneScan (6). The annotation of *A. radioresistens* strain SA188 resulted in 2,741 ORFs, seven rRNAs, 65 tRNAs, and 33 ncRNAs.

The analysis of the SA188 genome revealed the presence of multiple enzymes involved in salinity tolerance, oxidative stress, and drought tolerance. The genome encodes isochorismate synthase (EC: 5.4.4.2) (7) and trehalose phosphatase (EC: 3.1.3.12) (8), which increases salinity and drought tolerance, as well as α,α-trehalose-phosphate synthase (EC: 2.4.1.15), which confers multiple stress protection in plants (9, 10) and improvement of drought tolerance in potato (11). The genome of SA188 harbors genes encoding different enzymes allowing plants to tolerate glyphosate herbicides such as 3-phosphoshikimate 1-carboxyvinyltransferase (EC: 2.5.1.19) (12, 13) and protoporphyrinogen oxidase (EC: 1.3.3.4) (14–16). Further analysis of the SA188 genome sequence will help to elucidate the metabolic pathways involved in plant growth–promoting interaction and its molecular mechanism.

### Accession number(s).

The genome of *A. radioresistens* strain SA188 was deposited at DDBJ/EMBL/GenBank under the accession number LWGP00000000. The version described in this paper is the first version, LWGP01000000.
